# A Circulating Exosome RNA Signature Is a Potential Diagnostic Marker for Pancreatic Cancer, a Systematic Study

**DOI:** 10.3390/cancers13112565

**Published:** 2021-05-24

**Authors:** Yixing Wu, Hongmei Zeng, Qing Yu, Huatian Huang, Beatrice Fervers, Zhe-Sheng Chen, Lingeng Lu

**Affiliations:** 1 Department of Endocrinology, Second Affiliated Hospital of Guangzhou Medical University, Guangzhou 510260, China; wyxingz@126.com; 2 National Central Cancer Registry, National Cancer Center/National Clinical Research Center for Cancer/Cancer Hospital, Chinese Academy of Medical Sciences and Peking Union Medical College, Beijing 100730, China; hongmeizeng2011@163.com; 3 Center for Cancer and Blood Disorders, Children’s National Medical Center, Washington, DC 20010, USA; qyu@cnmc.org; 4 Department of Imaging, Guizhou Qianxinan People’s Hospital, Xingyi 652400, China; huanght3702321@126.com; 5 Département Prévention Cancer Environnement, Centre Léon Bérard—Université Lyon 1, 69008 Lyon, France; beatrice.fervers@lyon.unicancer.fr; 6 UMR Inserm 1296 “Radiations: Défense, Santé, Environnement”, Centre Léon Bérard, 69008 Lyon, France; 7 Department of Pharmaceutical Sciences, St. John’s University, New York, NY 11439, USA; chenz@stjohns.edu; 8 Department of Chronic Disease Epidemiology, Yale School of Public Health, School of Medicine, New Haven, CT 06520, USA; 9 Center for Biomedical Data Science, Yale University, 60 College Street, New Haven, CT 06520, USA; 10 Yale Cancer Center, Yale University, 60 College Street, New Haven, CT 06520, USA

**Keywords:** exosome, early detection, KRAS mutation, pancreatic cancer, RNA signature

## Abstract

**Simple Summary:**

Most patients with pancreatic cancer are diagnosed at an advanced stage due to the lack of tools with high sensitivity and specificity for early detection. Aberrant gene expression occurs in pancreatic cancer, which can be packaged into nanoparticles (also known as exosomes or nano-sized extracellular vesicles) and then released into blood. In this study, we aimed to evaluate the diagnostic value of a circulating exosome RNA signature in pancreatic cancer. Our findings indicate that the circulating exosome RNA signature is a potential marker for the early detection or diagnosis of pancreatic cancer.

**Abstract:**

Several exosome proteins, miRNAs and *KRAS* mutations have been investigated in the hope of carrying out the early detection of pancreatic cancer with high sensitivity and specificity, but they have proven to be insufficient. Exosome RNAs, however, have not been extensively evaluated in the diagnosis of pancreatic cancer. The purpose of this study was to investigate the potential of circulating exosome RNAs in pancreatic cancer detection. By retrieving RNA-seq data from publicly accessed databases, differential expression and random-effects meta-analyses were performed. The results showed that pancreatic cancer had a distinct circulating exosome RNA signature in healthy individuals, and that the top 10 candidate exosome RNAs could distinguish patients from healthy individuals with an area under the curve (AUC) of 1.0. Three (*HIST2H2AA3*, *LUZP6* and *HLA-DRA*) of the 10 genes in exosomes had similar differential patterns to those in tumor tissues based on RNA-seq data. In the validation dataset, the levels of these three genes in exosomes displayed good performance in distinguishing cancer from both chronic pancreatitis (AUC = 0.815) and healthy controls (AUC = 0.8558), whereas a slight difference existed between chronic pancreatitis and healthy controls (AUC = 0.586). Of the three genes, the level of *HIST2H2AA3* was positively associated with *KRAS* status. However, there was no significant difference in the levels of the three genes across the disease stages (stages I–IV). These findings indicate that circulating exosome RNAs have a potential early detection value in pancreatic cancer, and that a distinct exosome RNA signature exists in distinguishing pancreatic cancer from healthy individuals.

## 1. Introduction

Live cell-secreted bilayer membranous extracellular nano-sized vesicles (also known as exosomes) carry bioactive macromolecules of proteins, DNA, RNA, lipids and metabolites that are exported out of and mirror their cells of origin. The exosome process was initially thought to be a mechanism utilized by cells for getting rid of ‘wastes’ that are toxic to them, favoring growth, malignant phenotypes or the avoidance of immune surveillance [1]. Accumulating evidence shows that exosomes play important roles in cell-to-cell communications through the transfer of bioactive donor-cell molecules to recipient cells, leading to physiological changes in the recipients and promoting tumor growth [2,3], and by which the donor cells may increase the number of their partners to orchestrate together against “unfavorable” environments [4]. Exosomes are secreted by living cells, and are frequently found in various body fluids, e.g., blood, saliva and urine [5,6]. These properties facilitate exosome collection, enabling the monitoring of disease progression and the response to treatment through minimally or non-invasive liquid biopsies. Furthermore, abnormal alterations in cells of origin can be found earlier by analyzing exosomes than by analyzing necrotic cell-derived products, such as circulating cell-free DNA/RNAs, which usually occurs at later disease stages. In addition, larger numbers of exosomes are secreted by tumor cells than by normal cells [7,8], increasing the abundance of tumor-derived exosomes and making tumor information more detectable. Accumulating studies have shown the potential for the utilization of exosomes in the diagnosis/early detection, prognosis and monitoring of the treatment of human cancer, as well as for the engineering of vehicles to treat human cancer [9,10].

Pancreatic ductal adenocarcinoma (PDAC) and closely-related variants comprise the major type (>90%) of pancreatic cancer with a continuously rising incidence in the United States [11,12]. PDAC affects more than 45,000 individuals and leads to over 38,000 deaths each year in the United States [12]. In 2020, the incident cases of pancreatic cancer were 495,773 worldwide, and the number of deaths was increased approximately 2.38-fold compared to year 1990 (466,003 vs. 196,000) [13]. Patients diagnosed at early stages with pancreatic cancer show considerable progress in treatment. Unfortunately, most patients still present with advanced disease, and less than 20% have resectable tumors [14,15,16], suggesting that no tools are available for early detection or diagnosis with sufficient sensitivity and specificity.

Pancreatic malignancy is a comprehensive consequence of genetic and epigenetic events. Hundreds of somatic mutations occur in PDAC with the highest frequency of 90% found in *KRAS* [17,18]. Animal models show that mutant KRAS^G12D^ or KRAS^G12V^ is sufficient to initiate the development of PanINs, which progress to invasive metastatic PDAC after sufficient latency [19,20]. The presence of mutated *KRAS* DNA in both cell-free DNA and exosomes from PDAC suggests the potential of *KRAS* in the early detection of PDAC [21,22]. Recently, Yang et al. reported exosome *KRAS* DNA mutation in about 40% of pancreatic cancer patients [23]. Allenson et al. reported 66% *KRAS* DNA mutations in pancreatic cancer patients vs. 7.4% healthy controls [24]. Although some exosome proteins (CD44v6, Tspan8, EpCAM and CD104) in combination with several circulating exosome miRNA candidates have also shed light on the diagnosis of PDAC [22,25], these panels do not have enough sensitivity and specificity for the early detection of PDAC. More efforts are needed in order to explore novel markers for the detection of PDAC. Thus, the purpose of this study was to investigate circulating exosome RNAs as a potential marker of pancreatic cancer detection.

## 2. Results

### 2.1. Differential Expression of Circulating Exosome RNAs between Pancreatic Cancer Patients and Healthy Controls

**Figure 1:**
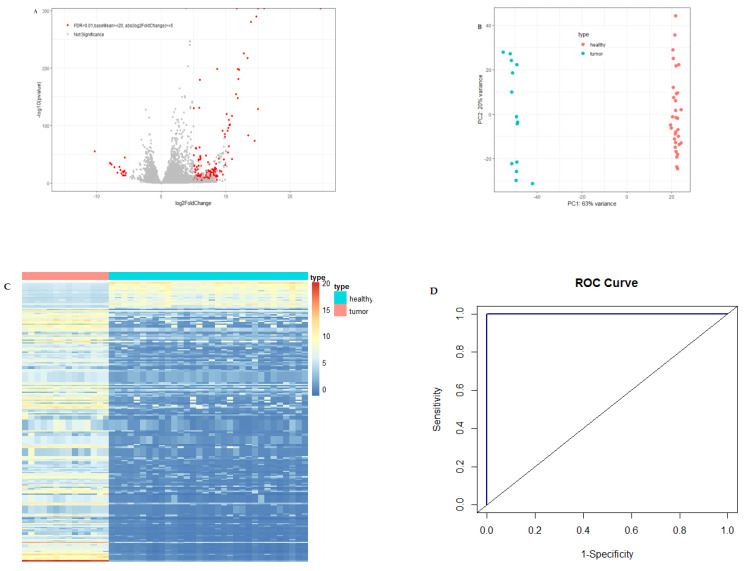
Differential expression of circulating exosome RNAs in pancreatic cancer patients and healthy individuals. (**A**) Volcano plot of differential circulating exosome RNAs. The red dots represent significant genes with baseMean ≥20, the absolute value of log2FoldChange ≥5 and FDR <0.01, and the gray dots represent non-significant results. (**B**) PCA plot of circulating exosome RNAs showing the distance between the individuals. Light-coral dots represent healthy individuals and turquoise dots represent patients with pancreatic cancer. (**C**) Heatmap of significantly differential circulating exosome RNAs for patients with pancreatic cancer (light-coral bar) and healthy individuals (turquoise bar). The columns of the heatmap represent individuals, and the rows represent circulating exosome RNA genes. (**D**) Receiving operating characteristic (ROC) curve with the exosome RNAs of 10 candidate genes. AUC is the area under the curve.

A large number of differentially expressed circulating exosome RNAs were identified in pancreatic cancer patients from the Shanghai study, and the fold-changes for the genes with significant differential expression are shown in Figure 1A.

Figure 1B is the PCA plot of RNA-seq data for circulating exosome RNAs. Pancreatic cancer patients showed a distinct circulating exosome RNA signature obtained from healthy individuals. The two groups were completely separated, particularly with regard to the first component, with a 63% variance, and each of the groups was clustered exclusively. The heatmap again shows that pancreatic cancer patients had a distinct circulating exosome RNA pattern in comparison to healthy individuals (Figure 1C).

To evaluate the performance of differential circulating exosome RNAs in distinguishing the health status of either pancreatic cancer patients or healthy controls, we used a random forest model and constructed an ROC curve with the normalized counts of the top 10 among the significantly differentially expressed genes (Figure 1D). The 10 circulating exosome RNAs showed a large area under the curve (AUC), with a value of 1.0 in distinguishing the patients from healthy controls.

To better understand the biological relevance of the differentially expressed RNAs in circulating exosomes derived from pancreatic cancer patients, we further performed IPA analysis based on the database released on 21 November 2018. The main results of IPA are shown in Table 1. 

**Figure 2:**
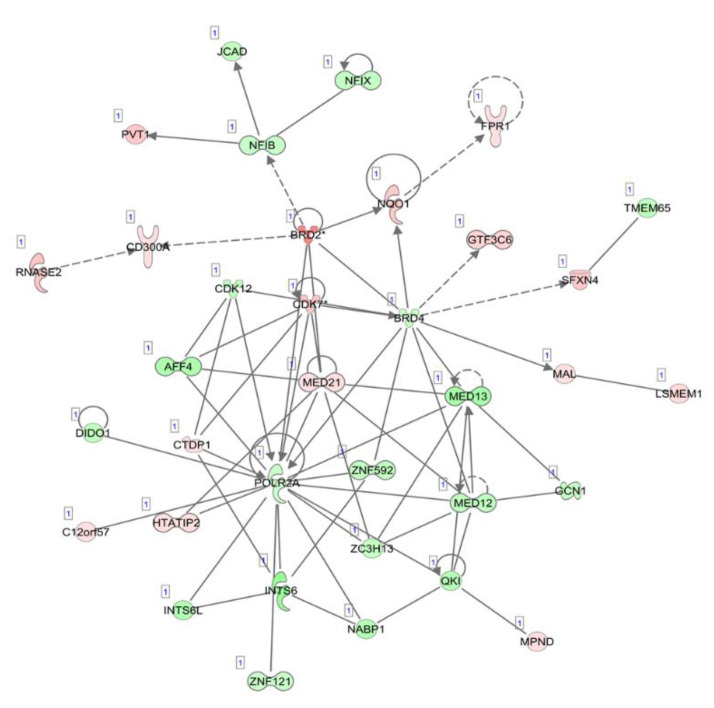
Illustration of the top network of differentially expressed transcripts, related to “gene expression”, “RNA post-transcriptional modification” and “neurological diseases” in circulating exosome RNAs, compared between pancreatic cancer patients and healthy individuals. Red and green shading indicate the up- and downregulation of transcripts in circulating exosomes derived from patients with pancreatic cancer relative to healthy controls, respectively, with the color intensity corresponding to the degree of fold-change. Solid and dotted lines indicate direct and indirect relationships, respectively.

The top five canonical pathways were enriched, which included oxidative phosphorylation, mitochondrial dysfunction, the sirtuin signaling pathway, estrogen receptor signaling and the protein ubiquitination pathway. The top diseases and disorders were listed as cancer, organismal injury and abnormalities, infectious diseases, endocrine system disorders and gastrointestinal disease. The differentially expressed circulating exosome RNAs were statistically significantly enriched in five major characteristics of molecular and cellular functions, which included gene expression, cell death and survival, RNA post-transcriptional modification, protein synthesis and the cell cycle. Network analyses demonstrated several downregulated gene expression-related molecules of nuclear factors, zinc finger and RNA polymerases, an upregulated oncogene, PVT1, and dysregulated cell cycle-dependent kinases (Figure 2).

### 2.2. Differential Expression of Candidate Genes in Pancreas Tumors vs. Normal Tissues and Their Associations with Disease Stage

**Figure 3:**
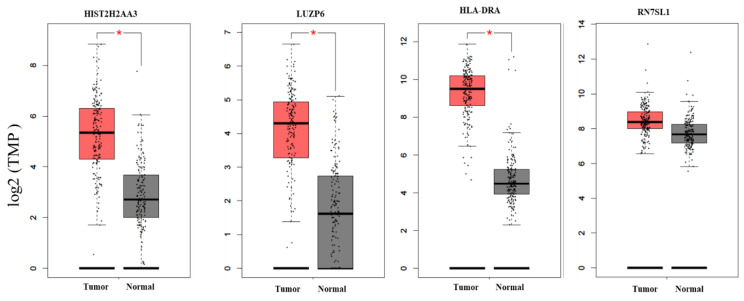
Boxplots of the candidate gene expression levels in pancreatic adenocarcinoma and normal pancreas tissues. The vertical (*y*) axis is the gene expression level of transcripts per million (TPM) in log2 (TPM +1), and the horizontal (*x*) axis is the status of tissues (left box = tumor (*n* = 179) and right box = normal (*n* = 171)). The solid line inside the box is the median and the box edges are the 25th and 75th quartiles (interquartile range, IQR). The whiskers are 1.5 × IQRs. The black dots are the expression levels of the gene in individuals. * *p* < 0.01.

**Figure 4:**
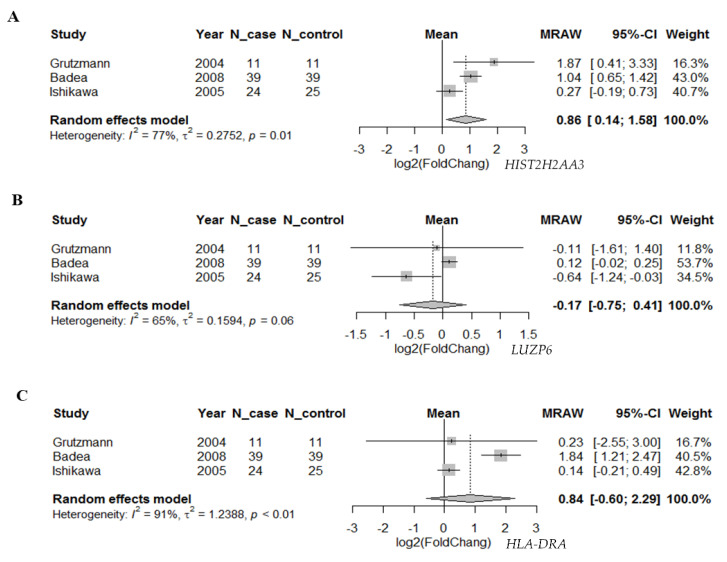
Forest plot of the differential gene expression in primary pancreatic cancer. Random-effects meta-analysis results of the fold change in log2 for *HIST2H2AA3* (**A**), *LUZP6* (**B**) and *HLA-DRA* (**C**) in primary pancreatic ductal adenocarcinoma vs. normal pancreas tissues.

To further investigate whether the differential expression pattern found in circulating exosomes would be reproducible in pancreatic tumors vs. normal tissues, we searched two databases for the 10 candidate genes. First, we compared the expression levels of these genes based on the RNA-seq data in TCGA pancreatic adenocarcinoma (*n* = 179) and matched normal tissue plus the genotype-tissue expression in pancreas tissues (*n* = 171) using GEPIA (Figure 3). We found that there were statistically significant differences in the three genes *HIST2H2AA3*, *LUZP6* and *HLA-DRA* comparing between pancreatic tumors and normal tissues (*p* < 0.01), with higher levels in tumors vs. normal tissues. The differences in the other seven genes were not statistically significant, although *RN7SL1*, *MIR663AHG*, *GPM6A* and *FAM184B*, for example, were higher in tumors vs. normal tissues (*p* > 0.01). Then, we retrieved gene expression array data from Oncomine^®^ for the three coding genes *HIST2H2AA3*, *LUZP6* and *HLA-DRA*. Random-effects meta-analysis results showed that the levels of *HIST2H2AA3* showed a significant difference between pancreatic cancer and normal pancreas tissues, but those of *LUZP6* and *HLA-DRA* did not (Figure 4). The fold change in log2 was 0.86-fold (95% CI: 0.14–1.58, *p* = 0.019) for *HIST2H2AA3*, −0.17-fold (95% CI: −0.75–0.41, *p* = 0.57) for *LUZP6* and 0.84-fold (95% CI: −0.60–2.29, *p* = 0.25) for *HLA-DRA*, respectively.

**Figure 5:**
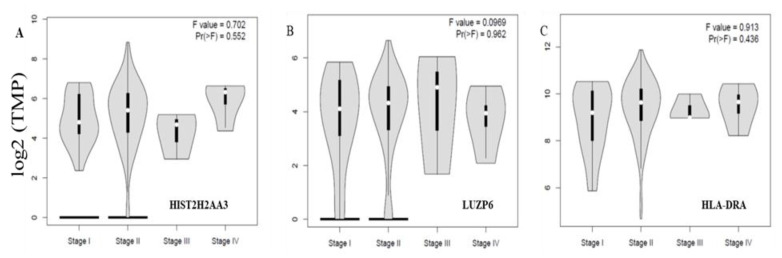
Violin plots of the three gene expression levels across the disease stages in pancreatic cancer. The vertical axis is the gene expression level of transcript per million (TPM) in log2 (TPM +1) for *HIST2H2AA3* (**A**), *LUZP6* (**B**) and *HLA-DRA* (**C**), respectively, and the horizontal axis is the disease stage. The violin shape is the frequency distribution of the gene expression levels; the inside boxplot represents the median (white dot), interquartile range (the box edge) and 95% confidence interval (the solid black line).

Finally, we further examined whether or not the three differential genes in tumors vs. normal tissues were associated with disease stages. Figure 5 shows the levels of the three genes *HIST2H2AA3, LUZP6* and *HLA-DRA* across the disease stages. None of the three was significantly associated with the disease stage in pancreatic cancer (*p* > 0.05).

### 2.3. Association of Candidate Gene Expressions with KRAS Mutation Status in Pancreas Tumors

**Figure 6:**
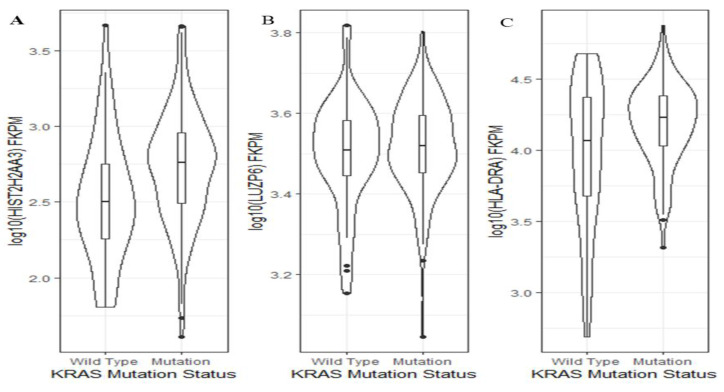
Violin plots of the three gene expression levels and *KRAS* mutation status in pancreatic cancer. The vertical axis is the gene expression level in log10 (FKPM) for *HIST2H2AA3* (**A**), *LUZP6* (**B**) and *HLA-DRA* (**C**), respectively, and the horizontal axis is the *KRAS* mutation status. The violin shape is the frequency distribution of gene expression levels; the inside boxplot represents the median (black line), interquartile range (the box edge) and 95% confidence interval (the solid black line).

Activating *KRAS* mutations are present in over 90% of PDAC cases and are found in increasing frequency in developing PanIN lesions [19,26]. Thus, we wanted to further explore whether or not the levels of the three genes were *KRAS* mutation status-dependent. Using TCGA pancreatic cancer data, we found that between *KRAS* wild-type and mutants, there was a significant difference in the *HIST2H2AA3* expression level (*p* = 0.001), but not in that of *LUZP6* (*p* = 0.5) or *HLA-DRA* (*p* = 0.06) (Figure 6). Patients with *KRAS* mutation had a higher *HIST2H2AA3* expression level than those with the wild-type gene (2.7 ± 0.38 vs. 2.5 ± 0.42, log10 (FKPM)).

### 2.4. Performance Validation of the HIST2H2AA3, LUZP6 and HLA-DRA Signature

**Figure 7:**
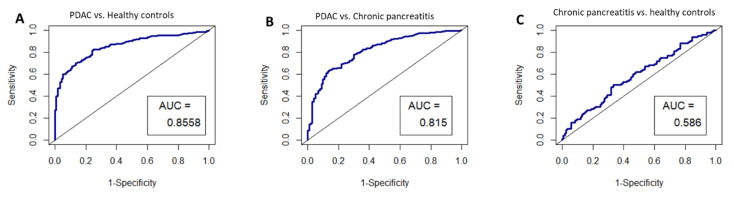
ROC curves for the circulating exosome RNA signature of *HIST2H2AA3*, *LUZP6* and *HLA-DRA* in pancreatic cancer patients and healthy controls; (**A**) pancreatic cancer vs. healthy controls; (**B**) pancreatic cancer vs. chronic pancreatitis; and (**C**) chronic pancreatitis vs. healthy controls, respectively. AUC is the area under the curve for the accuracy of the signature distinguishing the two groups.

Given that *HIST2H2AA3*, *LUZP6* and *HLA-DRA* expression levels were found to be independent of the disease stage, and that they were found to be statistically different between patients and healthy controls in both exosomes and tissues, we chose these three genes for performance validation in an independent study dataset. The ROC curves were constructed by creating logistic models, and the results are illustrated in Figure 7. The AUCs were 0.8558 (95% CI: 0.82–0.89) for pancreatic cancer vs. healthy controls, 0.815 (0.77–0.86) for pancreatic cancer vs. chronic pancreatitis, and 0.586 (0.51–0.66), respectively.

## 3. Discussion

In this study, we evaluated the potential of circulating exosome RNAs as detection biomarkers in pancreatic cancer using publicly accessed datasets. We found that patients with pancreatic cancer had a distinct circulating exosome RNA signature in comparison to healthy controls. Ten of the top differentially expressed genes (five upregulated and five downregulated RNAs) were capable of distinguishing patients from healthy controls with an excellent performance (AUC = 1.0). We also found that three (*HIST2H2AA3*, *LUZP6* and *HLA-DRA*) of these 10 differential circulating exosome RNAs had a significantly higher level in tumors vs. normal tissues based on the RNA-seq data, suggesting that the pattern of these three genes in circulating exosomes was reproducible in tumor tissues. Using the levels of the three genes (*HIST2H2AA3*, *LUZP6* and *HLA-DRA)* in exosomes as a signature, we obtained a high sensitivity and specificity (accuracy) in distinguishing patients with pancreatic cancer from healthy controls and from those with chronic pancreatitis in an independent study.

Exosomes are bilayer membrane nanosized vesicles (sized from 30–100 nm in diameter) that are actively secreted by live cells, which carry the contents of RNAs, DNAs, proteins and lipids from the cells of origin. Several studies have shown the discrepancies in RNA contents between the cells of origin and exosomes [27,28,29,30,31], suggesting that the bioactive molecular contents of exosomes are enriched. During exosome production, both microenvironment factors (such as stimuli and cell state) and RNA motifs may affect the compositions of RNAs in exosomes [32,33,34,35,36,37]. In addition to the relative abundance of small RNAs in exosomes, long non-coding RNAs (lncRNAs) and mRNAs are also enriched. Of the 10 candidate differential exosome RNAs, RN7SL1 and miR663AHG are lncRNAs. The potential of exosome lncRNAs as biomarkers and gene regulators has been demonstrated [38,39], which even at a low number of copies in the cells are more prone to be enriched in exosomes [27,40]. Thus, it is not surprising that the genes had differential expression in exosomes but not in cells. Similarly, Perez-Boza and colleagues also reported that mRNAs were enriched with more unique or exclusively mRNA genes in exosomes than in cells [27]. The coding mRNAs in exosomes could remain active, and once they are taken, could promote chemotherapy resistance in recipient cells [41]. Taken together, the uneven distribution of RNA species and fragments in exosomes derived from cells suggests that the delivery of RNAs into exosomes is selective. RNAs with some structural motifs (e.g., ACCAGCCU, CAGUGAGC and UAAUCCCA) are preferentially recognized by RNA-binding protein YB-1 and RNA methyltransferase NSUN2, which likely act as mediators, sorting specific mRNAs into exosomes [32]. However, the mechanism(s) underlying the selective packaging of RNAs in exosomes remains a challenge in the study of extracellular vesicles.

Interestingly, the top five enriched pathways for the differentially expressed RNAs in circulating exosomes were consistent with the findings of previous studies, which reported that these pathways were abnormal in pancreatic cancer. Metabolic reprogramming and mitochondrial dysfunction have been reported in pancreatic cancer, and by enhancing the nucleotide biosynthesis, the frequently mutated *KRAS* gene stimulates cancer cell proliferation [42,43,44,45]. Targeting metabolic reprogramming pathways is a potential therapeutic strategy in the management of pancreatic cancer [42,44,46,47]. As a lysine deacetylase, sirtuin 1 is involved in the regulation of gene expression, such as that of *TP53*, and promotes the development of pancreatic cancer in cooperation with *KRAS* mutations [48]. Previous studies have demonstrated that reproductive factors and hormone usage increased the risk of pancreatic cancer, and that a high level of estrogen receptors elevates the mortality risk of the disease [49,50,51,52,53]. However, we found that on average, male PDAC patients survived 3.5 months longer than female patients (median: 21.4 (95% CI: 19.4–37.1) for men vs. 17.9 (15.8–24.1) months for women, respectively), although the difference did not reach statistical significance (log-rank *p*-value = 0.37) in this study (Supplementary Materials, Figure S1). A multivariate Cox model also excluded a significant association between sex and mortality in our previous study [54]. This was most likely because the sample size in this study was too small to replicate the general report that a higher mortality rate is observed in males than females [13]. An estrogen signaling system also exists in men, which plays an important role in the regulation of biological and pathological processes in both men and women [55,56]. Seeliger and colleagues reported no significant association between sex and estrogen signaling in PDAC [53]. However, they demonstrated significantly higher survival in patients with a low level of estrogen receptors in PDAC than those with a high level of estrogen receptors [53]. In addition, a recent study reported that ubiquitination signaling could activate *KRAS* and promote macropinocytosis in pancreatic cancer [57]. Moreover, *H. pylori* infection is a risk factor for pancreatic cancer [58,59,60,61], which is also in agreement with the finding of infectious disease in the top diseases and disorders.

It has been reported that RN7SL1 inhibited the translation of TP53 by competing with HuR for binding to the 3′-UTR of *TP53*, consequently leading to cell cycle progression and suppressing cellular senescence and autophagy [62]. When unshielded RN7SL1 in circulating exosomes was taken up by the immune cells, it incited an inflammatory response by activating the PRR RIG-I and promoted tumor growth, metastasis and therapy resistance [63]. *miR663AHG* is a host gene of miR-663, which has been shown to be significantly downregulated in pancreatic cancer [64]. The overexpression of miR-663 led to attenuated proliferation and invasion by directly targeting eEF1A2 [64]. *HIST2H2AA3* and *HIST1H4K* are histone gene variants, and have been shown to be dysregulated in human cancer [65]. *HIST2H2AA3* was downregulated in N-nitrosodienthylamine-induced hepatocellular carcinoma [66], whereas it was upregulated in pancreatic cancer [67], suggesting that the aberrant *HIST2H2AA3* expression in tumors was tissue-specific. In agreement with previous observations, we found that the level of *HIST2H2AA3* in both exosome and cell compartments was higher in patients with pancreatic cancer than in healthy controls.

Immune escape is a hallmark of human cancer, including pancreatic cancer, and is a process in which many mechanisms are involved, such as the loss or downregulation of antigen presentation, immune checkpoint-induced CD8+ T cell exhaustion and the loss of tumor infiltration lymphocytes (“cold” tumors). Pandha and colleagues reported that the downregulation of MHC-I molecules was observed in pancreatic cancer, whereas HLA-DRA in MHC-II molecules was upregulated [68]. The findings in this study support the previous results beyond the trafficking of MHC-II HLA-DR molecules in exosomes [69].

Little is known about the biological relevance of leucine zipper protein 6 (LUZP6). The *LUZP6* gene is located at chromosome 7, and its encoded protein is a putative tumor-self antigen. It may elicit an immune response in individuals with myeloproliferative disease who have received interferon alpha [70]. Another study showed that the LUZP6 protein was positive in the majority of glioblastoma [71], suggesting that it may also be related to tumorigenesis. Interestingly, *LUZP6* RNA is enriched in salivary exosomes and, together with another three genes (*IL1R2*, *VPS4B* and *CAP1*) comprising an RNA signature, could distinguish high from low insulin resistance as an extracellular RNA marker [72].

Although the level of *HIST2H2AA3,* rather than that of *LUZP6* and *HLA-DRA,* in tumor tissues was positively associated with *KRAS* mutation, there was no significant difference in the level of *HIST2H2AA3*, *LUZP6* or *HLA-DRA* expression across the disease stages. This finding suggests that these markers may be more reliable than the frequency of *KRAS* mutations in the early detection of pancreatic cancer, given that the frequency of *KRAS* mutations increases with the disease stages. Moreover, in three independent gene expression array studies with a relatively small sample size, the differential expression of *HIST2H2AA3* was reproducible in the tumors vs. normal pancreas tissues, but those of *LUZP6* and *HLA-DRA* were not.

There are some limitations in this study. One is that the plasma samples were collected from patients who presented clinical manifestations of the disease in the Shanghai study. Another is the fact that no disease stage information or other demographic information was available for the patients and healthy controls in the circulating exosome RNA analyses in the Shanghai study. In addition, smoking status was also unavailable in the Shanghai study, which is considered to be an established risk factor for pancreatic cancer [73]; thus, we cannot evaluate how smoking status affects the RNA molecules in circulating exosomes. However, the findings of this study emphasize the value of further investigating circulating exosome RNAs as potential markers in pancreatic cancer detection. Longitudinal studies should be conducted to further evaluate this method’s sensitivity and specificity, as well as its positive and negative predictive values (PPV, NPV) for disease screening, particularly in individuals at high risk [74,75].

## 4. Materials and Methods

### 4.1. Data Sources

Two Gene Expression Omnibus (GEO) datasets (GSE100232 and GSE100206) for the discovery of exosome RNA signatures and one GSE133684 dataset for validation were retrieved from the National Center for Biotechnology Information (NCBI) (https://www.ncbi.nlm.nih.gov) (11 March 2019 for GSE100232 and GSE100206 and 12 March 2021 for GSE133684). The methods used for exosome isolation from plasma biospecimens, exosome identification and RNA sequencing for these three GEO datasets have been described as elsewhere [76,77,78]. Briefly, using an ultracentrifuge, exosomes were isolated from plasma samples, followed by size and morphology characterization using transmission electron microscopy and exosome surface marker CD63 detection using Western blotting. Total RNAs from each isolated exosome sample were purified using Trizol reagent (Invitrogen, Waltham, MA, USA). After DNase I treatment, RNA-seq libraries were prepared and RNA sequencing was performed using an Illumina sequencing platform. GSE100232 contains RNA-seq data from 14 patients with pancreatic cancer, and GSE100206 contains data from 32 healthy individuals [76,77]. GSE133684 consisted of data from 284 pancreatic cancer patients, 100 chronic pancreatitis patients and 117 healthy controls [78].

The Cancer Genome Altas (TCGA) and Genotype-Tissue Expression (GTEx) RNA-seq data were also used to compare the expression of genes in pancreatic cancer vs. normal tissues using Gene Expression Profiling Interactive Analysis (GEPIA2) (http://gepia2.cancer-pku.cn) (27 March 2019). The demographic information on the patients in TCGA data has been described elsewhere previously [54]. The differential expressions of candidate genes, which were determined by whole genome expression arrays, were retrieved from the gene expression dataset Oncomine^TM^ (https://www.oncomine.org) (28 March 2019) (ThermoFisher Scientific Inc., Waltham, MA, USA) using the parameters of differential analysis (cancer vs. normal), cancer type (pancreatic ductal adenocarcinoma), sample type (clinical specimen), data type (mRNA) and gene (e.g., *HIST2H2AA3*) and total subjects ≥20.

### 4.2. Statistical and Bioinformatics Analyses

Statistical analyses were performed using R package 3.5 (https://www.r-project.org) (21 March 2020). The differential expression of circulating exosome RNAs, compared between pancreatic cancer patients and healthy individuals, was analyzed using the DESeq2 package, in which the negative binomial distribution was applied. Principal component analysis (PCA) was performed to obtain the top 2 components with the largest variances, after data transformation and batch effect removal, for PCA plot visualization. Heatmaps were constructed based on the significant differential expression genes with adjusted *p*-values greater than 0.01, and the means of normalized counts of all samples (baseMean) ≥20 FPKM (fragments per kilobase of transcript per million mapped reads), and the absolute value of log2FoldChange ≥5. The top 10 candidate genes (5 upregulated genes, consisting of *HIST2H2AA3*, *HIST1H4K*, *HLD-DRA*, *RN7SL1* and *LUZP6*, and 5 downregulated genes consisting of *FAM184B*, *FGF23*, *NEUROD2*, *miR663AHG* and *GPM6A* in the circulating exosome RNAs of pancreatic cancer patients) with significant differential expression were selected for receiving operating characteristic (ROC) curve analysis using the random forest algorithm, in which the sensitivity and specificity were calculated. In the validation stage, a logistic model was used for the construction of ROC curves, using *HIST2H2AA3*, *LUZP6* and *HLA-DRA* mRNA levels in exosomes. Ingenuity pathway analysis (IPA) (Qiagen Bioinformatics, Redwood City, CA, USA) was performed for the differentially expressed genes with adjusted *p*-values < 0.01, and baseMean ≥ 20 FPKM, and the absolute value of log2FoldChange ≥2 (to include more genes in the IPA analysis).

A random-effects model in the meta-analysis was performed to determine the fold change in gene expression array-based differential gene expression compared between pancreatic cancer and normal tissues, following a methodology that has been previously described elsewhere [79,80]. A *p-*value of less than 0.05 was considered statistically significant if not specified.

## 5. Conclusions

This study revealed a distinct exosome RNA signature in plasma, which was capable of distinguishing pancreatic cancer patients from healthy individuals. The top 10 candidate exosome RNAs showed a high performance in the diagnosis of pancreatic cancer. Of these, three upregulated genes (*HIST2H2AA3*, *LUZP6* and *HLA-DRA*) in exosomes also showed the same pattern in pancreatic tumors vs. normal pancreas tissue. There were no significant differences in the levels of these three genes across the disease stages, although *HIST2H2AA3* expression was associated with *KRAS* mutation status. These findings suggest that circulating exosome RNAs are potential markers in the detection of pancreatic cancer. Further independent studies with a relatively large sample size and the inclusion of individuals with non-cancer diseases (e.g., chronic pancreatitis), or conducted in populations at high risk of pancreatic cancer, are warranted.

## Figures and Tables

**Table 1:** Summary of ingenuity pathway analysis (IPA) results.

Name	*p*-Value	Overlap Genes
Top Canonical Pathways		
Oxidative Phosphorylation	4.11 × 10^−18^	51/109
Mitochondrial Dysfunction	6.04 × 10^−17^	65/171
Sirtuin Signaling Pathway	4.20 × 10^−15^	88/292
Estrogen Receptor Signaling	1.25 × 10^−13^	51/134
Protein Ubiquitination Pathway	4.39 × 10^−11^	75/271
Top Diseases and Disorders		
Cancer	3.73 × 10^−6^–1.06 × 10^−62^	2644
Organismal Injury and Abnormalities	3.73 × 10^−6^–1.06 × 10^−62^	2681
Infectious Diseases	3.61 × 10^−7^–1.47 × 10^−29^	487
Endocrine System Disorders	3.09 × 10^−6^–2.55 × 10^−27^	2081
Gastrointestinal Disease	3.09 × 10^−6^–5.50 × 10^−23^	2243
Top Molecular and Cellular Functions		
Gene Expression	1.12 × 10^−7^–1.97 × 10^−27^	701
Cell Death and Survival	4.29 × 10^−6^–1.40 × 10^−22^	961
RNA Post-Transcriptional Modification	6.14 × 10^−9^–9.99 × 10^−19^	143
Protein Synthesis	3.93 × 10^−7^–9.13 × 10^−18^	389
Cell Cycle	3.75 × 10^−6^–1.83 × 10^−17^	523

## Data Availability

All data used in this work are publicly accessible as described in the materials and Methods.

## Supplementary Materials

The following are available online at https://www.mdpi.com/article/10.3390/cancers13112565/s1, Figure S1: Kaplan–Meier overall survival curves stratified by sex in pancreatic cancer. Male patients showed superior overall survival in comparison with female patients. The median survival values were 21.4 (95% CI: 19.4–37.1) months for men and 17.9 (95% CI: 15.8–24.1) months for women, respectively. On average, male patients survived approximately 3.5 months longer than female patients. However, the difference was not statistically significant (log-rank *p*-value = 0.37).
