# Supplementary material for: A Circulating Exosome RNA Signature Is a Potential Diagnostic Marker for Pancreatic Cancer, a Systematic Study
Source: Cancers (Basel). 2021 May 24;13(11):2565. doi: 10.3390/cancers13112565 (PMC8197236; doi:10.3390/cancers13112565)
Supplement: Supplementary file 1 [file cancers-13-02565-s001.zip › cancers-1221524-supplementary.pdf]

A Circulating Exosome RNA Signature is a Potential Diagnostic Marker for Pancreatic Cancer, a Systematic Study

Yixing Wu, Hongmei Zeng, Qing Yu, Huatian Huang, Beatrice Fervers, Zhesheng Chen and Lingeng Lu

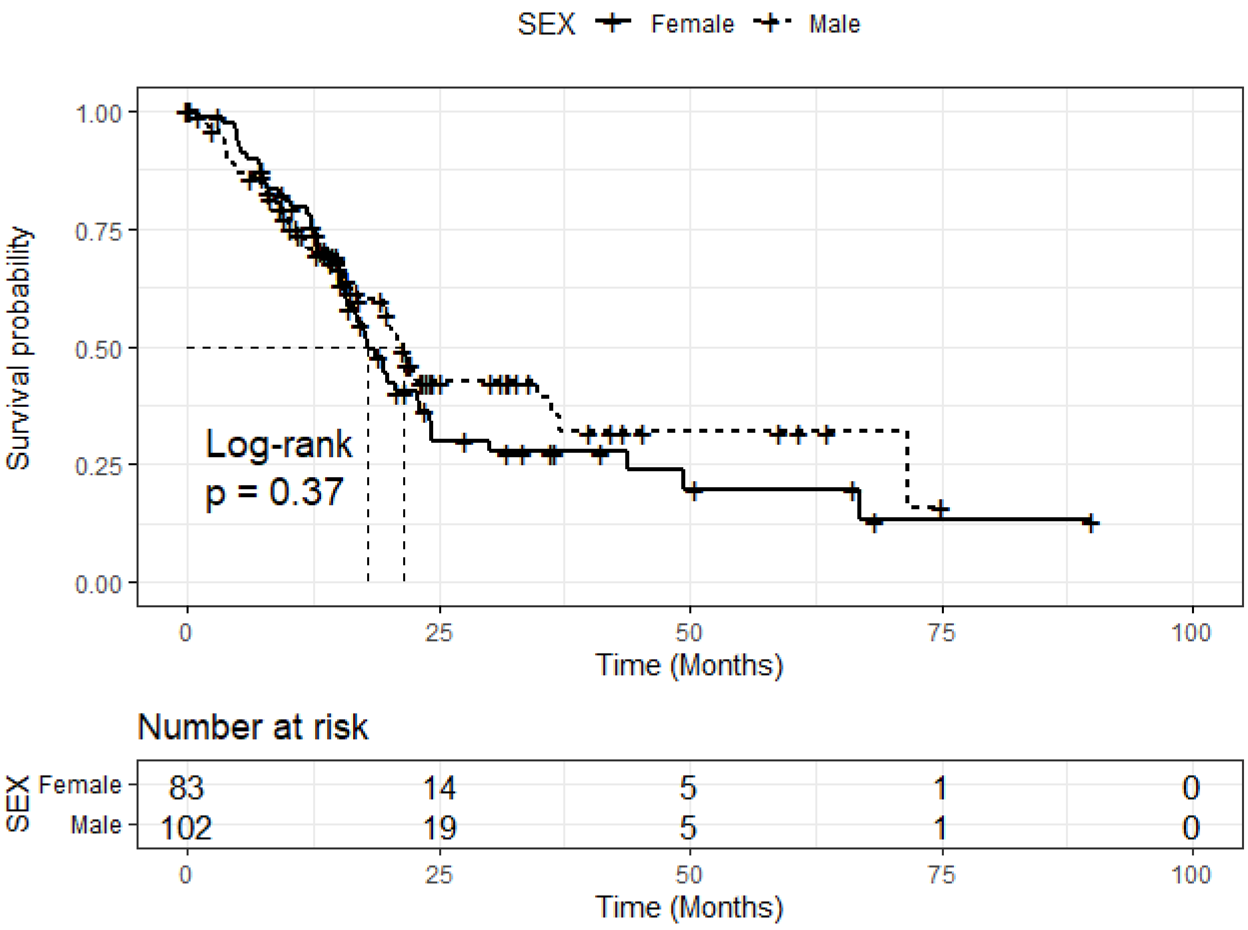

Figure S1. Kaplan-Meier overall survival curves stratified by sex in pancreatic cancer. Male patients showed a superior overall survival in comparison to female patients. The median survivals were 21.4 (95% CI: 19.4–37.1) months for men and 17.9 (95% CI: 15.8–24.1) months for women, respectively. On average, male patients survived approximately 3.5 months longer than female patients. However, the difference was not statistically significant (log-rank *p* value = 0.37).
